# High mobility group box 1-induced epithelial mesenchymal transition in human airway epithelial cells

**DOI:** 10.1038/srep18815

**Published:** 2016-01-07

**Authors:** Yu-Ching Chen, Sarah Statt, Reen Wu, Hao-Teng Chang, Jiunn-Wang Liao, Chien-Neng Wang, Woei-Cherng Shyu, Chen-Chen Lee

**Affiliations:** 1 Graduate Institute of Immunology, College of Medicine, China Medicine University, Taichung, Taiwan; 2 Center for Comparative Respiratory Biology and Medicine, Internal Medicine, College of Medicine, University of California at Davis, USA; 3 Institute of Basic Medical Science, College of Medicine, China Medicine University, Taichung, Taiwan; 4 Institute of Veterinary Pathology, College of Veterinary Medicine, National Chung Hsing University, Taichung, Taiwan; 5 Department of Microbiology and Immunology, School of Medicine, China Medicine University, Taichung, Taiwan; 6 Department of Health and Nutrition Biotechnology, Asia University, Taichung, Taiwan

## Abstract

Epithelial–mesenchymal transition (EMT) is implicated in bronchial remodeling and loss of lung function in chronic inflammatory airway diseases. Previous studies showed the involvement of the high mobility group box 1 (HMGB1) protein in the pathology of chronic pulmonary inflammatory diseases. However, the role of HMGB1 in EMT of human airway epithelial cells is still unclear. In this study, we used RNA sequencing to show that HMGB1 treatment regulated EMT-related gene expression in human primary-airway epithelial cells. The top five upregulated genes were *SNAI2*, *FGFBP1*, *VIM*, *SPARC (*osteonectin), and *SERPINE1*, while the downregulated genes included *OCLN*, *TJP1* (*ZO-1*), *FZD7*, *CDH1* (E-cadherin), and *LAMA5*. We found that HMGB1 induced downregulation of E-cadherin and ZO-1, and upregulation of vimentin mRNA transcription and protein translation in a dose-dependent manner. Additionally, we observed that HMGB1 induced AKT phosphorylation, resulting in GSK3β inactivation, cytoplasmic accumulation, and nuclear translocation of β-catenin to induce EMT in human airway epithelial cells. Treatment with PI3K inhibitor (LY294006) and β-catenin shRNA reversed HMGB1-induced EMT. Moreover, HMGB1 induced expression of receptor for advanced glycation products (RAGE), but not that of Toll-like receptor (TLR) 2 or TLR4, and RAGE shRNA inhibited HMGB1-induced EMT in human airway epithelial cells. In conclusion, we found that HMGB1 induced EMT through RAGE and the PI3K/AKT/GSK3β/β-catenin signaling pathway.

Epithelial mesenchymal transition (EMT) is a process through which epithelial cells that normally interact with basement membranes via their basal surface undergo multiple biochemical changes, causing them to differentiate into a mesenchymal-cell phenotype with enhanced migratory ability, invasiveness, and elevated resistance to apoptosis^1^,^2^. EMT is associated with embryo implantation, embryogenesis, organ development, and tissue regeneration; however, it has also been implicated in deleterious roles in chronic inflammation diseases, such as organ fibrosis, and in cancer progression and metastasis^1^. In chronic airway diseases, such as asthma and chronic obstructive pulmonary disease (COPD), EMT is involved in bronchial remodeling and loss of lung function^3^,^4^. Damaged airway epithelial cells that undergo EMT stimulate TGF-β production and increase collagen synthesis, as well as cell proliferation by airway fibroblasts and myofibroblasts^5^. This leads to subepithelial fibrosis and lamina reticularis thickening. Reducing E-cadherin expression in EMT also triggers epidermal growth factor receptor activation and subsequent expression of thymic stromal lymphopoietin (TSLP) and thymus and activation-regulated chemokine (TARC) in the airway epithelium, which imply Th2-related allergic inflammation^6^. This might dampen lung dysfunction, including bronchoconstriction, mucus production, and airway remodeling^7^,^8^. In severe asthma, EMT causes subepithelial fibrosis and thickening of the bronchial wall^9^. In COPD, EMT causes bronchial fibrosis and reticular basement-membrane fragmentation in the large and small airways, which is associated with disease severity^10^. Therefore, causes of airway structure changes constitute key hallmarks of airway remodeling^11^,^12^. Airway remodeling has been implicated in corticosteroid resistance, which hampers drug delivery to parenchymal lung tissue and contributes to reduced response to steroid treatment in severe asthma or COPD patients and unwanted side effects resulting from prolonged high-dose steroid therapy^13^,^14^.

The high mobility group box 1 (HMGB1) protein exhibits dual functions as a nuclear protein that regulates gene expression and nucleosome stability^15^ and as a cytokine, which stimulates the release of pro-inflammatory cytokines, such as tumor necrosis factor (TNF) and interleukin (IL)-1α, IL-6, and IL-8, in monocytes^16^, macrophages^17^, and neutrophils^18^ during inflammation. Previous studies have showed that HMGB1 participates in the pathology of chronic pulmonary inflammatory diseases, such as asthma^19^ and COPD^20^ and that HMGB1 levels were positively associated with the severity of those diseases,. We previously reported HMGB1 involvement in allergen-induced airway remodeling in a chronic asthma mouse model^21^, and its induction of myofibroblast differentiation in primary human-lung fibroblasts^22^. Given the importance of HMGB1 in airway remodeling during chronic airway inflammatory diseases, we hypothesized that HMGB1 may directly influence airway epithelial cells by inducing EMT. Therefore, in this study, we cultured human airway epithelial cells to determine the effect of HMGB1 on EMT and the mechanisms involved. We found that HMGB1 induced mesenchymal transition in airway epithelial cells through the receptor for advanced glycation products (RAGE) and the β-catenin signaling pathway.

## Results

### HMGB1 induces EMT-related gene expression in human airway epithelial cells

**Figure 1:**
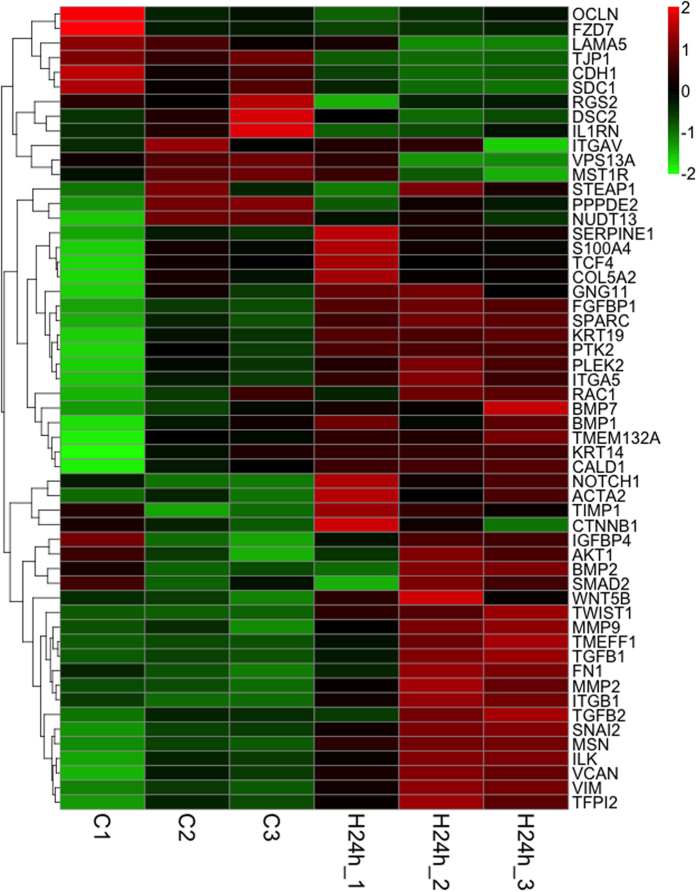
Heat map of EMT-related gene expression in HMGB1-treated human primary airway epithelial cells. Human primary airway epithelial cells were treated with HMGB1 (300 ng/mL) for 24 h and mRNA was extracted for analysis of EMT-related gene expression by RNA-sequencing assay. Red and green represent upregulation and downregulation, respectively. Three different clones of primary airway epithelial cells were used, which were isolated from different samples of human tracheobronchial tissues.

**Figure 2:**
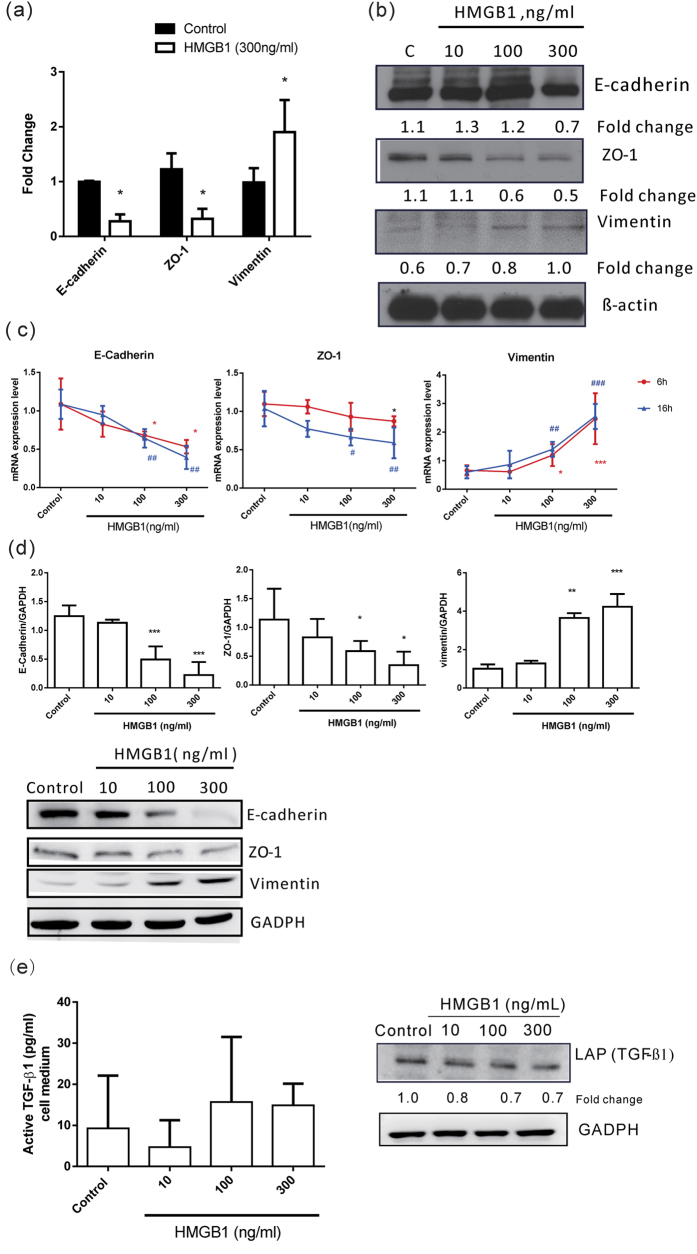
HMGB1-induced EMT in human airway epithelial cells. Human primary airway epithelial cells and BEAS-2B cells were treated with different doses of HMGB1 for mRNA analysis at different time points, and at 24 h for protein analysis. E-cadherin, ZO-1, and vimentin mRNA expression was detected by real-time quantitative PCR in (**a**) human primary airway epithelial cells and (**c**) in BEAS-2B cells after HMGB1 treatment (300 ng/mL) for 24 h. E-cadherin, ZO-1, and vimentin protein expression in (**b**) human primary airway epithelial cells andBEAS-2B cells (**d**) was assessed by western blot analysis. Data are expressed as mean ± SD (*n* = 3–5). *p < 0.05, **p < 0.01, ***p < 0.001, as compared with the control group. (**e**) TGF-β1 expression in cell lysate (LAP) was assessed by western blot analysis and in cell medium (active form) by ELISA. Quantification of protein expression was performed using ImageJ software. Data are expressed as mean ± S.D. (n = 4). In immunoblotting assay, gels have been run under the same experimental conditions. Then cropped blots were incubated with different primary antibodies for analysis of EMT markers expression. Original blots of (**b**) are presented in Supplementary Fig. 3.

First, we assessed whether HMGB1 induces EMT in human primary-airway epithelial cells by RNA-sequencing analysis. As shown in Fig. 1 and Table 1, we analyzed 55 EMT-related genes^23^,^24^. Compared to the control groups, HMGB1-treated cells showed significant upregulation of 22 genes and downregulation of 10 genes (*p* < 0.05; Table 1). The top five upregulated genes were *SNAI2*, *FGFBP1*, *VIM*, *SPARC* (osteonectin), and *SERPINE1*, and the downregulated genes included *OCLN*, *TJP1* (*ZO-1*), *FZD7*, *CDH1* (E-cadherin), and *LAMA5*. When airway epithelial cells undergo EMT, they show decreased expression of epithelial markers, such as the junction proteins occludin, ZO-1, and E-cadherin, and increased expression of mesenchymal markers, including vimentin and fibronectin^4^. RNA-sequencing analysis showed that HMGB1 decreased occludin, ZO-1, and E-cadherin expression, and increased vimentin and *SNAI2* expression in human primary-airway epithelial cells, indicating that HMGB1 might induce EMT in these cells. Next, we used real-time polymerase chain reaction (PCR) to confirm the changes in ZO-1, E-cadherin, and vimentin mRNA expression. As shown in Fig. 2A,C, HMGB1 downregulated E-cadherin and ZO-1 expression and upregulated vimentin mRNA expression in both human primary-airway epithelial cells and human bronchial epithelial cell lines (BEAS-2B cells) in a dose-dependent manner. Further investigation of protein expression showed the same trend as observed with mRNA transcription, wherein HMGB1 decreased E-cadherin and ZO-1 expression and increased vimentin protein expression in a dose-dependent manner in both human primary-airway epithelial cells (Fig. 2B) and BEAS-2B cells (Fig. 2D). Given their similar biological functions and high transfection efficiency, we chose BEAS-2B cells to analyze the mechanisms associated with HMGB1-induced EMT. Using immunocytochemistry, we verified that human airway epithelial cells expressed high levels of E-cadherin and ZO-1 and low levels of vimentin (Supplementary Figure S1), while treatment with HMGB1 (100 ng/mL and 30 ng/mL) resulted in loss of E-cadherin and ZO-1 expression and high levels of vimentin expression. TGF-β1 is a potent EMT inducer, and many inflammatory cytokines enhance TGF-β1-induced EMT in airway epithelial cells^25^,^26^,^27^. To clarify whether HMGB1-induced EMT might occur following TGF-β release, we searched for evidence of the active forms of TGF-β in culture medium and pro-form TGF-β in cell lysate. As shown in Fig. 2E,F, HMGB1 treatment did not induce TGF-β1 production.

### HMGB1 induces EMT through the Akt/GSK-3β/β-catenin pathway

**Figure 3:**
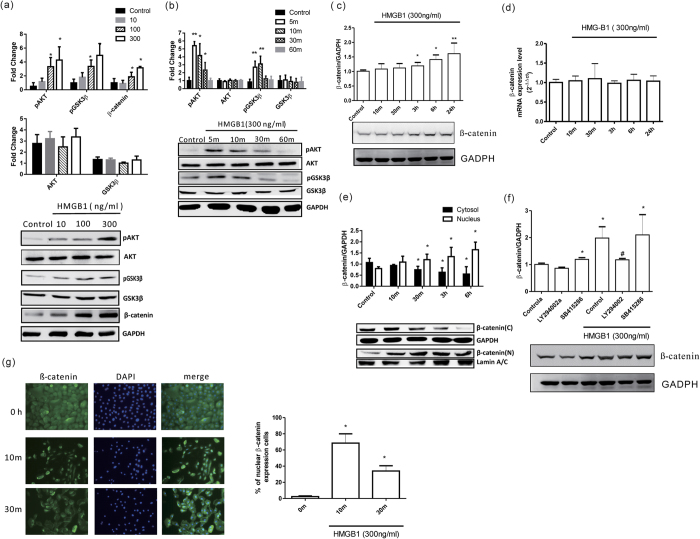
HMGB1-activated AKT/GSK3β/β-catenin signaling pathways. (**a**) BEAS-2B cells were treated with different doses of HMGB1 for 10 min and expression of different proteins was detected by western blot. (**b**) BEAS-2B cells were treated with HMGB1 (300 ng/mL) for different periods and analyzed for expression of different proteins by western blot. (**c**) BEAS-2B cells were treated with HMGB1 (300 ng/mL) for different periods and analyzed for β-catenin expression by western blot. (**d**) BEAS-2B cells were treated with HMGB1 (300 ng/mL) for different periods and analyzed for β-catenin mRNA expression by real-time quantitative PCR. (**e**) BEAS-2B cells were treated with HMGB1 (300 ng/mL) for different periods and analyzed for nuclear and cytosolic β-catenin expression by western blot. (**f**) BEAS-2B cells were treated with 10 μM PI3K inhibitor (LY294002) and GSK-3β inhibitor (SB415286) for 10 min, and then treated with HMGB1 (300 ng/mL) for 24 h. β-catenin expression was assessed by western blot analysis. Quantification of protein expression was performed using ImageJ software. Data are expressed as mean ± SD. *p < 0.05, **p < 0.01, as compared with the control group. #*p* < 0.05, as compared the HMGB1-treatment group. (A,B) (*n* = 4); (C–F) (*n* = 3). (**g**) Immunofluorescence staining of β-catenin (green) was detected by fluorescence microscopy. Data are expressed as mean ± SD (*n* = 4). *p < 0.05, as compared with the 0-min grou*p*. In immunoblotting assay, gels have been run under the same experimental conditions. Then cropped blots were incubated with different primary antibodies for analysis of signaling pathway.

Cell signaling involved in EMT includes the Ras/MAPK, PI3K/Akt, Smad, RhoB, and β-catenin pathways^28^. We found that HMGB1 induced AKT phosphorylation in human airway epithelial cells in a dose- (Fig. 3A) and time- (Fig. 3B) dependent manner. Given that glycogen synthase kinase-3 beta (GSK-3β) is a major downstream target of AKT, we examined whether HMGB1 affects GSK-3β activity. As shown in Fig. 3A,B, HMGB1 inhibited GSK-3β activity by increasing its phosphorylation^29^ at ser9 without affecting total GSK-3β expression. Phosphorylation and inactivation of GSK-3β prevents β-catenin degradation and results in cytosolic accumulation and eventual nuclear translocation of β-catenin. In the nucleus, β-catenin interacts with the T-cell factor (TCF)/lymphoid enhancer-binding factor (LEF) family, leading to transcription of genes that induce EMT^30^. Therefore, we hypothesized that HMGB1 might induce β-catenin nuclear translocation. HMGB1 treatment induced β-catenin accumulation and nuclear translocation in a time-dependent manner in BEAS-2B cells (Fig. 3C,E). HMGB1-induced β-catenin accumulation did not occur through increased mRNA transcription (Fig. 3D), but rather through post-transcriptional modification. Similar to results observed in the BEAS-2B cells, human primary airway epithelial cells (Fig. 3G) showed β-catenin activation and nuclear translocation after HMGB1 treatment.

**Figure 4:**
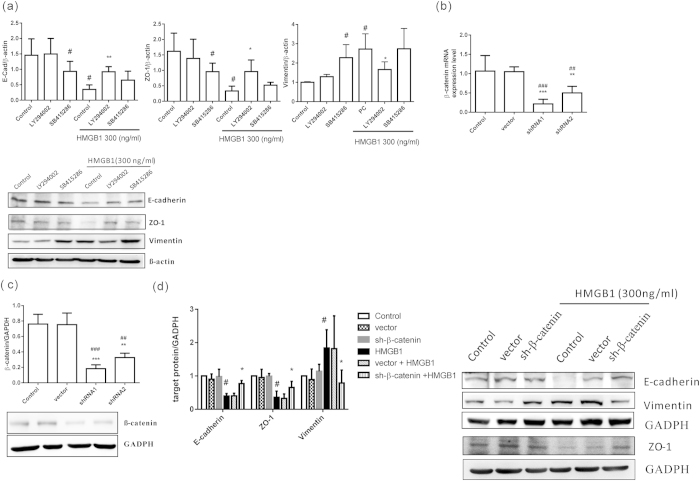
Inhibition of the AKT/GSK3β/β-catenin signaling pathway decreased HMGB1-induced EMT. (**a**) BEAS-2B cells were treated with 10 μM PI3K inhibitor (LY294002) and GSK-3β inhibitor (SB415286) for 10 min, and then treated with HMGB1 (300 ng/mL) for 24 h. Protein expression was detected by western blot analysis. Quantification of E-cadherin, ZO-1, and vimentin was performed using ImageJ software. Data are expressed as mean ± SD (*n* = 5). #*p* < 0.05, as compared with the control group. *p < 0.05, as compared with the HMGB1-treated group. (**b**) BEAS-2B cells were transduced with lentiviral-expressed β-catenin shRNA (1 MOI) for 72 h and selected by puromycin. The mRNA from stable clones expressing β-catenin-targeting shRNAs was analyzed by quantitative real-time PCR. Data are expressed as mean ± SD (*n* = 5). ##*p* < 0.01, ###*p* < 0.001, as compared with the control group. **p < 0.01, ***p < 0.001, as compared with the vector group. (**c**) β-catenin protein expression was detected by western blot analysis in a stable β-catenin shRNA BEAS-2B cell clone. Quantification of protein expression was performed using ImageJ software. Data are expressed as mean ± SD (*n* = 3). ##*p* < 0.01, ###*p* < 0.001, as compared with the control group. **p < 0.01, ***p < 0.001, as compared with the vector group. (**d**) A stable β-catenin shRNA BEAS-2B cell clone was treated with HMGB1 (300 ng/mL) for 24 h, and protein expression of E-cadherin, ZO-1, and vimentin was detected by western blot analysis. Quantification of protein expression was performed using ImageJ software. Data are expressed as mean ± SD (*n* = 4). #*p* < 0.05, as compared with the control group. *p < 0.05, as compared with the HMGB1 group. In immunoblotting assay, gels have been run under the same experimental conditions. Then cropped blots were incubated with different primary antibodies for analysis of signaling pathway.

Next, we used different inhibitors to confirm whether HMGB1 induced EMT through the PI3K/AKT, GSK-3β, and β-catenin signaling pathways (Figs 3F and 4). First, we determined whether HMGB1 induction of the PI3K/AKT and GSK-3β signaling pathways was dependent upon β-catenin activation. As shown in Fig. 3F, cells treated with a GSK-3β inhibitor (SB415286) without HMGB1 resulted in increased β-catenin expression. Additionally, following treatment with a PI3K inhibitor (LY294002), HMGB1-induced β-catenin expression was inhibited. However, a combined treatment with HMGB1 and the GSK-3β inhibitor did not show any synergistic effects on β-catenin expression. Next, we observed that HMGB1-induced E-cadherin and ZO-1 downregulation and vimentin upregulation were reversed when cells were treated with a PI3K inhibitor (Fig. 4A). When cells were treated with GSK-3β inhibitor, which stabilizes β-catenin, E-cadherin and ZO-1 expression decreased and vimentin expression increased in human airway epithelial cells, even in the absence of HMGB1. Similar to β-catenin expression, combined treatment with HMGB1 and the GSK-3β inhibitor did not show any synergistic effects on the expression of EMT markers. Next, the cells were transduced to express shRNAs targeting β-catenin. Of these, shRNA1-expressing cells showed significant knockdown of β-catenin mRNA and protein expression (Fig. 4B,C). In these cells, E-cadherin and ZO-1 downregulation and vimentin upregulation did not occur following HMGB1 treatment (Fig. 4D). Furthermore, β-catenin knockdown cells did not display changes in morphology or growth rate (Supplementary Figures S2C and S2D).

### HMGB1-induced EMT in human airway epithelial cells involves RAGE

**Figure 5:**
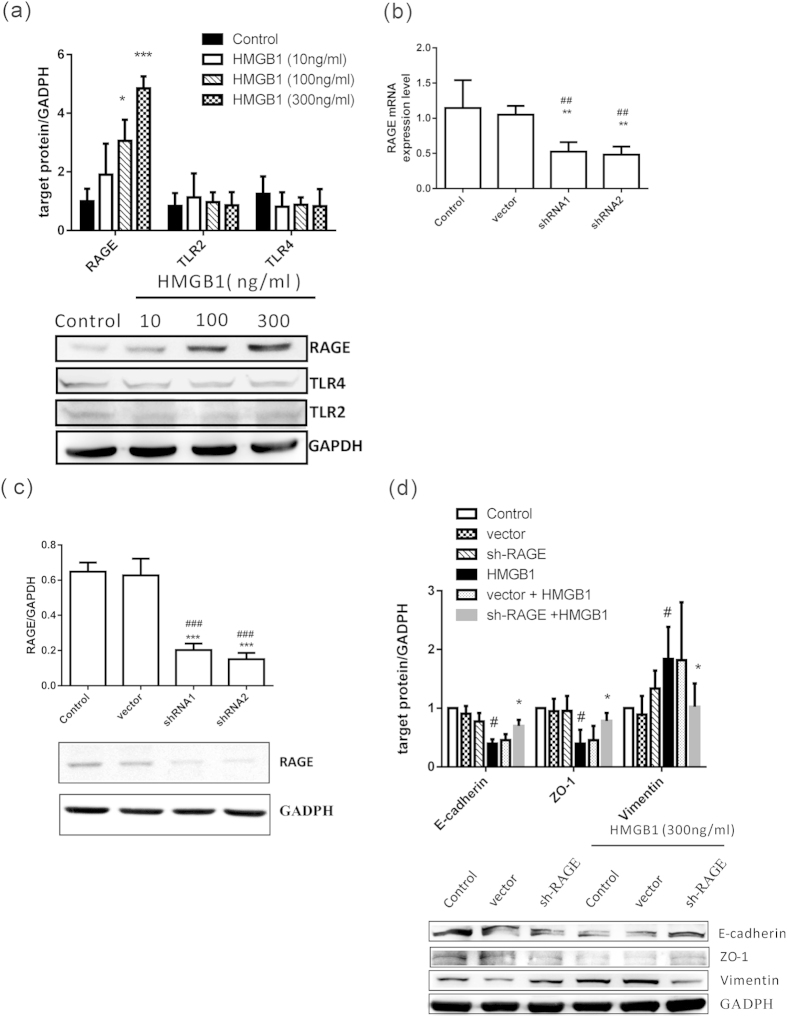
Induction of RAGE, but not TLR2 and TLR4 expression, in HMGB1-treated BEAS-2B cells. (**a**) BEAS-2B cells were treated with different doses of HMGB1 for 24 h to detect expression of HMGB1 receptors by western blot analysis. Quantification of protein expression was performed using ImageJ software. Data are expressed as mean ± SD (*n* = 4). *p < 0.05, ***p < 0.001, as compared with control group. (**b**) BEAS-2B cells were transduced with lentiviral-expressed RAGE shRNA (0.5 MOI) for 72 h and selected by puromycin. The mRNA from stable clones that expressed RAGE shRNA was detected by quantitative real-time PCR. Data are expressed as mean ± SD (*n* = 3). ##*p* < 0.01, as compared with the control group. **p < 0.01, as compared with the vector group. (**c**) RAGE protein expression was confirmed by western blot analysis in stably expressed RAGE shRNA BEAS-2B cells. Quantification of RAGE protein expression was performed using ImageJ software. Data are expressed as mean ± SD (*n* = 3). ###*p* < 0.001, as compared with the control group. ***p < 0.001, as compared with the vector group. (**d**) Stable clones expressing RAGE-targeting shRNAs (sh-RAGE) were treated with HMGB1 (300 ng/mL) for 24 h and assessed for E-cadherin, ZO-1, and vimentin protein expression by western blot analysis. Quantification of protein expression was performed using ImageJ software. Data are expressed as mean ± SD (*n* = 5). #*p* < 0.05, as compared with the control group. *p < 0.05, as compared with the HMGB1 group. In immunoblotting assay, gels have been run under the same experimental conditions. Then cropped blots were incubated with different primary antibodies for analysis of signaling pathway.

HMGB1 induces phagocyte activation and cell migration through binding to membrane receptors, such as RAGE, Toll-like receptor (TLR) 2, and TLR4, to activate downstream signaling pathways^31^,^32^,^33^. After treatment with HMGB1, we found that RAGE expression increased in a dose-dependent manner; however, TLR2 and TLR4 did not show any obvious changes (Fig. 5A). We silenced RAGE gene expression using shRNA (Fig. 5B,C) in order to investigate the role of RAGE in HMGB1-induced EMT. We found that RAGE knockdown blocked HMGB1-induced downregulation of E-cadherin and ZO-1 and upregulation of vimentin (Fig. 5D), suggesting that HMGB1 binding to RAGE activates downstream signaling pathways resulting in EMT induction. Additionally, HMGB1-induced β-catenin expression was inhibited by RAGE knockdown (Supplementary Figure S2B).

### HMGB1-induced cell migration through the RAGE, PI3K/AKT, GSK-3β, and β-catenin signaling pathways

**Figure 6:**
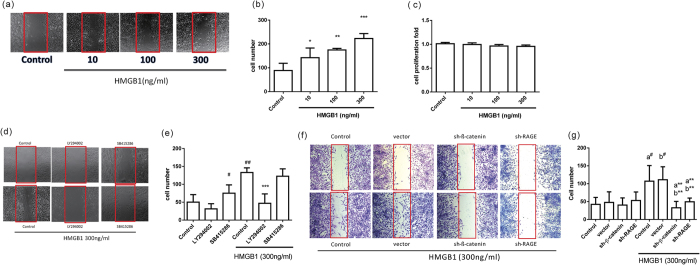
HMGB1 induced cell migration through activation of the RAGE/AKT/GSK3β/β-catenin signaling pathway. (**a**) BEAS-2B cells were treated with different doses of HMGB1 for 16 h, and cell migration was detected by *in vitro* scratch assay. (**b**) Quantification of cell migration was performed by counting the cells in the wound in four fields per sample. Data are expressed as mean ± SD (*n* = 5). *p < 0.05, **p < 0.01, ***p < 0.001, as compared with the control group, in which cells were treated with PBS. (**c**) BEAS-2B cells were treated with different doses of HMGB1 for 16 h, and cell proliferation was detected by BrdU-incorporation assay. Data are expressed as mean ± SD (*n* = 3). (**d**) Cells were pretreated with 10 μM of different inhibitors for 10 min, and then treated with HMGB1 (300 ng/mL) for 16 h. Cell migration was detected by *in vitro* scratch assay. (**e**) Quantification of cell migration was performed by counting the cells in the rectangle in four fields per sample. Data are expressed as mean ± SD (*n* = 5). #*p* < 0.05, ##*p* < 0.01, as compared with the control group. ***p < 0.001, as compared with the HMGB1 group. (**f**) Stable clones expressing shRNAs targeting β-catenin (sh-β-catenin) and RAGE (sh-RAGE) were treated with HMGB1 (300 ng/mL) for 16 h. Cell migration was detected by *in vitro* scratch assay. (**g**) Quantification of cell migration was performed by counting the cells in the red rectangle in four fields per sample. Data are expressed as mean ± SD (*n* = 4) a^#^*p* < 0.05, as compared with the control group. b^#^*p* < 0.05, as compared with the vector group. a**p < 0.05, as compared to the PC group. b**p < 0.05, as compared to the vector treated with HMGB1.

Since epithelial cells show increased mobility during EMT, we investigated the cell migration activity in human airway epithelial cells following HMGB1 treatment. As shown in Fig. 6A–C, HMGB1 induced cell migration in a dose-dependent manner, but did not affect cell proliferation which analyzed by BrdU incorporation assay (Fig. 6C) and Ki-67 expression (Supplementary Figure S1E). Treatment with PI3K inhibitor, β-catenin shRNA (sh-β-catenin), and RAGE shRNA (sh-RAGE) reduced HMGB1-induced cell migration (Fig. 6D–G). Treatment with GSK-3β inhibitor alone induced cell migration and did not show any synergistic effect when combined with HMGB1 treatment.

## Discussion

This study demonstrated that HMGB1 induced EMT, as well as cell migration, in human airway epithelial cells. In the airway, inflammation and growth factors constitute the primary factors associated with EMT induction and enhancement. Exogenous compounds, such as allergens and cigarette smoke, can induce EMT-related growth factors, such as TGF-β1, connective tissue growth factor, and platelet-derived growth factor^34^,^35^. Increased levels of cytokines, such as IL-1β and TNF-α, are also able to enhance TGF-β-induced EMT^25^,^26^,^27^. During inflammation, pro-inflammatory cytokines enhance TGF-β1-induced EMT, however, few reports indicate that pro-inflammatory cytokines are capable of inducing EMT in the absence of TGF-β1 involvement^36^. In this study, we found that HMGB1 by itself can induce EMT without inducing TGF-β1 production.

Increased HMGB1 levels were observed in COPD and asthma patients, especially in severe cases^19^,^37^. In our previous study, we found that HMGB1 was implicated in subepithelial fibrosis in a murine model of asthma^21^. Neutralization of lung HMGB1 using an HMGB1 antibody reduced allergen-induced collagen deposition around the airway and was accompanied by a decrease in TGF-β1 production. Here, we used cultured human bronchial epithelial cells and demonstrated that HMGB1 directly induced EMT through activation of the PI3K/AKT/GSK3β/β-catenin signaling pathway. β-catenin is an oncoprotein that plays an important role in EMT induction. In normal epithelium, β-catenin is associated with E-cadherin, and any free cytosolic β-catenin is phosphorylated by GSK3β and targeted for ubiquitin–dependent degradation^38^. We observed that treatment of airway epithelial cells with HMGB1 resulted in inactivation of GSK3β by PI3K-dependent AKT phosphorylation, which caused cytoplasmic accumulation of β-catenin and its subsequent translocation to the nucleus. Accumulation of cytosolic β-catenin following HMGB1 treatment was sustained for up to 24 h without any observable activation of β-catenin mRNA expression. This indicated that β-catenin activation by HMGB1 might not mediate transcriptional regulation. In the nucleus, β-catenin associates with TCF4 and acts as a transcriptional activator, resulting in expression of EMT-inducing transcription factors, such as ZEB1, ZEB2, SNAI1, SNAI2, Twist1, and Twist2^39^. Although we did not analyze the gene expression of all transcription factors (Table 1), we observed through RNA sequencing assays that HMGB1 treatment induced TCF4, SNAI2, and TWIST1 mRNA expression and increased Snail and Twist protein translation (Supplementary Figure S2A).

We also observed that GSK3β inhibition by HMGB1 was transient, whereas nuclear translocation and accumulation of β-catenin was sustained for up to 3h and 24 h, respectively. It is suggested that HMGB1 might activate other target molecules responsible for maintaining β-catenin activation and localization for extended periods. WNT signaling is an important inducer of β-catenin activation, and the WNT/β-catenin signaling pathway initiates a signaling cascade that is crucial during normal embryonic development, maintenance of adult tissue homeostasis, and in a variety of diseases, such as cancer, fibrosis, and neurodegeneration^40^. Previous studies showed that HMGB1 enhanced WNT/β-catenin signaling by increasing TCF/Lef1/β-catenin activity in zebrafish embryos; however, the exact mechanisms involved remain unclear^41^. Therefore, we examined whether HMGB1 treatment would regulate WNT-associated β-catenin activation in BEAS-2B cells. HMGB1 treatment induced low-density lipoprotein receptor-related protein 6 phosphorylation (LRP6-p) at Ser1490 in BEAS-2B cells for up to 24 h (Supplementary Figure S1D). LRP6 phosphorylation by GSK-3β and casein kinase 1 resulted in LRP6 recruitment of axin to the plasma membrane and activation of β-catenin^42^. Currently, the mechanism associated with HMGB1-induced LRP-6 activation is unknown and requires future study.

We observed through immunofluorescence staining in BEAS-2B cells that E-cadherin and ZO-1 were expressed in the cytoplasm and in the plasma membrane, while it was expected that E-cadherin and ZO-1 were predominantly localized to the plasma membrane (Supplementary Figure S1A). To confirm whether the result was due to cell-type differences, we verified E-cadherin expression and localization in human bronchial epithelial cell lines (HBE cells). Our results indicated that E-cadherin expressed and localized to both the plasma membrane and the cytoplasm, however, decreased E-cadherin expression was observed following HMGB1 treatment (300 ng/mL for 24 h; Supplementary Figure S1C). Previous studies have showed that E-cadherin was also expressed and localized to the cytoplasm in normal airway epithelial cells^43^,^44^. However, the reasons associated with ZO-1 and E-cadherin localization to the cytoplasm in BEAS-2B and HBE cells require further investigation.

Extracellular HMGB1 interacts with receptors, including RAGE, TLR2, and TLR4, to activate signaling pathways that cause chemotactic responses and the release of pro-inflammatory cytokines^31^,^32^,^33^. Of these receptors, RAGE is reportedly involved in HMGB1-induced cell migration and associated with lung fibrosis^45^. Here, we found that HMGB1 induced airway epithelial-cell migration and that EMT was primarily mediated by RAGE-HMGB1 interactions. Therefore, in addition to mediating fibrosis of the alveolar region, the RAGE-HMGB1 interaction is also involved in mesenchymal transition of airway epithelial cells, which might lead to subepithelial fibrosis. Downstream signaling pathways associated with RAGE include the Cdc42/Rac, MAPK/NF-κB, and PI3K/AKT pathways, all of which are involved in cell mobility^46^,^47^,^48^. In this study, we found that HMGB1 interaction with RAGE transduced PI3K/AKT activation to induce cell migration and EMT. Further investigation is needed to determine whether HMGB1-induced cell migration and EMT involves the Cdc42/Rac and/or MAPK/NF-κB signaling pathways.

In severe asthma, dysregulated bronchial epithelium is exhibits abnormally high levels of proliferation without sufficient compensation by death signaling, which results in thickening of the epithelium and lamina reticularis and contributes to airway remodeling^49^. HMGB1 has been identified as an activator of airway structure alteration through cell proliferation based on previous studies reporting that HMGB1 is involved in proliferation of lung smooth muscle cells, vascular endothelial cells, and fibroblasts, implicating it in pulmonary artery and airway remodeling in acute lung injury and in chronic airway inflammatory diseases^50^,^51^,^52^,^53^. However, whether HMGB1 causes airway epithelial cell proliferation remains unclear. In our study, we found that HMGB1 did not induce human airway epithelial cell proliferation.

**Figure 7:**
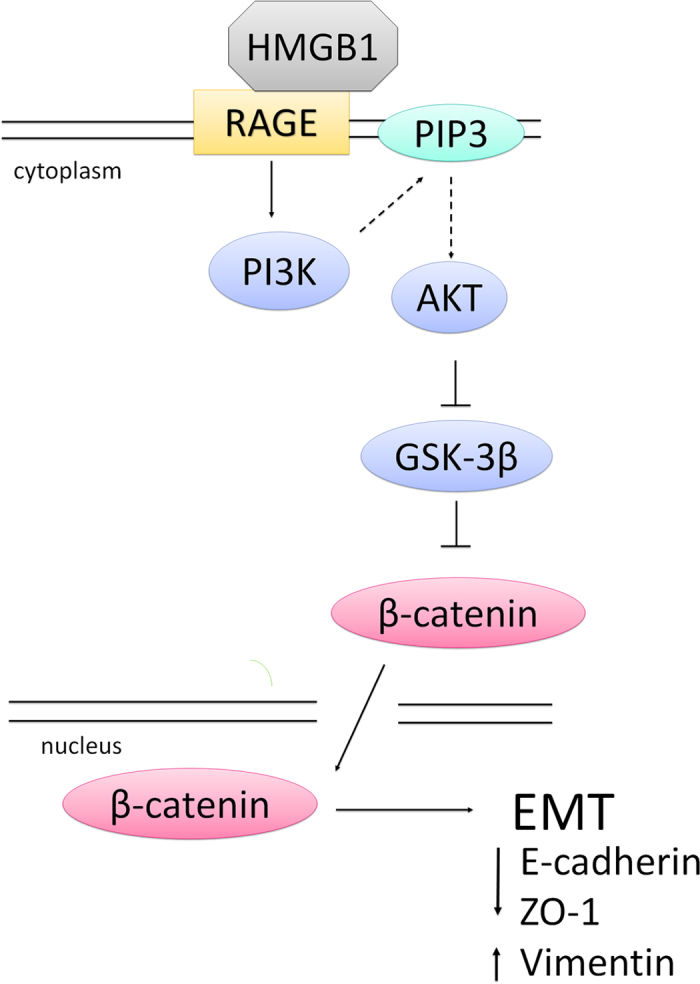
A model of HMGB1-induced EMT in human airway epithelial cells. HMGB1 binds RAGE to induce PI3K activation and subsequent AKT phosphorylation, GSK-3β inhibition, and β-catenin accumulation in the cytosol. β-catenin translocates to the nucleus and causes downregulation of ZO-1 and E-cadherin and upregulation of vimentin, thus inducing EMT and cell migration.

In conclusion, this study elucidated the role of HMGB1 in EMT in normal human airway epithelial cells (Fig. 7). EMT is an important mechanism involved in cancer cell migration and metastasis. Recently, in chronic airway diseases, such as COPD and asthma, EMT was associated with disease severity, steroid resistance, and lung dysfunction. Since steroids do not affect EMT, new agents are required to target EMT in patients with severe asthma and COPD. Our study indicated that HMGB1, a late-phase cytokine, might be a potential therapeutic target for treatment of severe cases of asthma and COPD in the future.

## Methods

### Cell culture

Primary normal human airway epithelial cells and immortalized normal human bronchial epithelial cell line (HBE cells) were kindly provided by Dr. Reen Wu (University of California-Davis, USA). Primary cells and culture conditions were prepared according to previous studies^54^. Human tracheobronchial tissues were obtained from the University of California Medical Center (Sacramento, CA, USA) with patient consent and from the National Disease Research Interchange (Philadelphia, PA, USA). The University Human Subjects Review Committee approved all procedures involved in tissue procurement. The methods were carried out in accordance with the approved guidelines. Human primary airway epithelial cells were cultured in Bronchial Epithelial Cell Growth Medium (BEGM) (Lonza Group Ltd. Basel, Switzerland). The culture conditions for HBE1 was described in previous studies^54^. The HBE cell line (BEAS-2B) was obtained from ATCC (Manassas, VA, USA) and maintained in Roswell Park Memorial Institute (RPMI) 1640 medium supplemented with 10% fetal bovine serum (FBS).

### Recombinant protein

Recombinant human HMGB1 was purified by *Escherichia coli* expression system to homogeneity as previously described^55^. Briefly, pET28a-HMGB1 expression plasmids were kindly provided by Dr. Wen-Cheng Xiong, Department of Neurology, Medical College of Georgia (Georgia Regents University, Augusta, GA, USA). We PCR-amplified HMGB1 from human primary macrophage cDNA and inserted the amplified segment into the vectors. The plasmids were transformed into *E. coli* BL21(DE3) pLysS cells (Novagen, San Diego, CA, USA), which were screened on Luria-Bertani (LB) plates containing 100 μg/mL carbenicillin (MDBio, New Taipei City, Taiwan) and 50 μg/mL chloramphenicol (MDBio), and then a single colony was inoculated in the same liquid medium at 37 °C overnight. The following day, the bacteria were subcultured in LB broth with the same concentration of the two antibiotics at 37 °C. When the OD_600_ value of the bacteria culture approached 0.8, the culture was moved to a 25 °C incubator and 0.5 mM of isopropyl β-D-1-thiogalactopyranoside added. After a 16-h induction, the bacteria were harvested by centrifuge and suspended in 20 mM HEPES (pH 7.9), 500 mM NaCl, 1 mM benzamidine, and 1 mM phenylmethylsulfonyl fluoride. The cell suspension was sonicated, and the soluble fraction isolated by centrifugation at 12,000 × *g* (4 °C) for 30 min. Recombinant HMGB1 was purified using an ÄKTA Prime system with a HisTrap FF column (GE Health, Uppsala, Sweden). The purified HMGB1 protein was verified by Coomassie blue staining after sodium dodecyl sulfate polyacrylamide gel electrophoresis (SDS-PAGE) analysis to verify purity >90%. To avoid endotoxin contamination, the purified HMGB1 protein was cleaned with EndoTrap (Hyglos GmbH, Germany). Activity of the recombinant human HMGB1 was determined by induction of TNF-α secretion in RAW 264.7 mouse monocyte cells. We were unaware of the post-translational modification of recombinant HMGB1. The dosages of HMGB1 (10, 100, and 300 ng/mL) used in this study were according to previous work by Watanabe *et al*.^19^, which observed HMGB1 concentrations in induced sputum at ranges of 0–590 ng/mL (91.5 ng/mL median) in asthmatic patients and 250–600 ng/mL in severely asthmatic patients. Zhou *et al*.^56^ showed that HMGB1 concentrations in sputum were 246–356 ng/mL in asthmatic patients. Ferhani *et al*.^20^ showed that HMGB1 concentrations in bronchial lavage fluid were 20–160 ng/mL in COPD patients. Therefore, HMGB1 might reach concentrations of 100 ng/mL and 300 ng/mL in the lung, especially in cases of severe asthma.

### Knockdown of gene expression by shRNA

Lentiviral expressed shRNAs were purchased from the National RNAi Core Facility Platform, Taiwan. Target sequences were as follows: β-catenin, TCTAACCTCACTTGCAATAAT (shRNA1) and TTGTTATCAGAGGACTAAATA (shRNA2); RAGE, CGAGTCCGTGTCTACCAGATT (shRNA1) and GCGGCTGGAATGGAAACTGAA (shRNA2). As described previously^22^, cells were infected with 1 multiplicity of infection (MOI) of lentiviral expressed β-catenin shRNA and 0.5 MOI of lentiviral expressed RAGE shRNA for 72 h. Stable clones of shRNA-infected cells were selected by culturing in 0.5 μg/mL puromycin.

### Cell proliferation assay

BEAS-2B cells were cultured with various concentrations of HMGB1 for 18 h and pulsed with 5-Bromo-2-deoxyuridine (BrdU) for an additional 6 h. Cell proliferation was determined by BrdU incorporation assay according to manufacturer instructions (Roche Diagnostics GmbH, Roche Applied Science, Germany)^57^.

### Secreted active TGF-β1 assay

Cells were treated with different doses of HMGB1 in RPMI medium contained with 1% FBS for 20h and collected medium for active TGF-β1 measurement by enzyme-linked immunosorbent assay (ELISA) according to manufacturer’s instructions (R&D Systems, Minneapolis, MN, USA).

### Cell migration assay

Cell migration was determined using the scratch assay. Briefly, cells were seeded in onto a 12-well plate and incubated to confluence. A cell-free wound area was then created by scratching the cell layer with a pipette tip. Cells were treated with different concentrations of HMGB1 and allowed to migrate into the cell-free wound in RPMI containing 1% FBS for 16 h.

### Immunofluorescence staining of EMT markers

Immunofluorescence staining of cellular protein was performed as described previously^58^. Briefly, cells cultured on cover slips were treated with different concentrations of HMGB1 for 16 h. Cells were fixed with 4% paraformaldehyde and stained using anti-E-cadherin Ab (Abcam, Cambridge, UK; UK Catalog No: ab40772), anti-ZO-1 Ab (Abcam; Catalog No: ab59720), and anti-vimentin (Sigma Aldrich, St. Louis, MO, USA; Catalog No: V6389) antibodies, while 4′,6-diamidino-2-phenylindole was used for nuclear staining (Calbiochem, San Diego, CA, USA). Cells were then incubated with a secondary antibody, Alexa Fluor 488-conjugated goat anti-mouse IgG (Jackson ImmunoResearch Laboratories Inc., Amish, PA, USA). Stained cells were imaged using a fluorescence and confocal microscope (Zeiss: Carl Zeiss, Göttingen, Germany). Fluorescence intensity was quantified using ImageJ software (http://imagej.nih.gov/ij/). The methods used to quantify the fluorescence density involved three steps: 1) count the fluorescence mean density, 2) divide the cell numbers, and 3) divide the control mean.

### RNA sequencing assay

Total RNA was extracted from cells using TRIzol reagent (Invitrogen, Carlsbad, CA, USA) according to manufacturer instructions. RNA integrity was determined using the RNA6000 Nano kit on the Agilent 2100 Bioanalyzer (Agilent, Santa Clara, CA, USA). RNA libraries were prepared using the low-throughput protocol of the Illumina TruSeq RNA sample preparation kit (Illumina, San Diego, CA, USA). Briefly, 1 μg of total RNA was used for poly-A mRNA selection, and selected mRNA was then fragmented in high-salt and high-temperature conditions. cDNA synthesis, end-repair, A-tailing, multiplex indexing, and PCR amplification was performed and the resulting libraries were quantified and analyzed on a HiSeq2000 (Illumina) for SR50. The resulting reads were aligned to the Hg19 transcriptome. Data analysis was completed using DESeq version 1.6.1^59^.

### Quantitative real-time PCR

RNA was converted into cDNA and then measured by quantitative real-time PCR using ABI PRISM 7900 Sequence Detector (Applied Biosystems, Foster City, CA, USA). The PCR product was amplified using SyBr Green regents. Statistically significant increases in target genes were determined using the threshold cycle (Ct) normalized with GADPH, and the expression level was reported as fold change using the 2-ddCT method^60^. The primer sequences used were as follows: E-cadherin, CCTGGGACTCCACCTACAGA (forward) and TGGATTCCAGAAACGGAGGC (reverse); ZO-1, GCCTCTCAACAGAAAGCAGAA (forward) and TGCCTCATCATTTCCTCGGG (reverse); vimentin, GACAATGCGTCTCTGGCACGTCTT (forward) and AAGAACCTGCAGGAGGCAGAAGAA (reverse); GADPH, TGCACCACCAACTGCTTAGC (forward) and TCTTCTGGGTGGCAGTGATG (reverse).

### Nuclear-extract preparation

After incubation with HMGB1 (300 ng/mL) for different periods, cells were washed twice with ice-cold phosphate-buffered saline (PBS). Cells were then scraped from the dishes and nuclear protein was extracted using NE-PER nuclear and cytoplasmic extraction kit according to manufacturer recommendations (Pierce, Rockford, IL, USA).

### Western blot analysis

The protein concentration of cell and tissue lysates was determined using a Bio-Rad protein assay kit (Bio-Rad, Richmond, CA, USA) according to manufacturer instructions. Immunoblotting was performed as described previously. Proteins were separated by SDS-PAGE, which was performed on 10% polyacrylamide gels, and then transferred onto polyvinylidene difluoride membranes (PerkinElmer Life Sciences, Boston, MA, USA) using the Panther^TM^ Semidry Electroblotter (Owl Scientific, Portsmouth, NH, USA). The blot was incubated overnight with blocking solution (5% skim milk) at 4 °C and then incubated with different primary antibodies (1:1000 dilution), including anti-E-cadherin Ab (Abcam), anti-ZO-1 Ab (Abcam), anti-vimentin (Sigma Aldrich), anti-Akt1-phospho (pS473) (Abcam; Catalog No: 2118-1), Anti-Akt (Abcam; Catalog No: 1085-1), anti-GSK-3β Phospho (Abcam; Catalog No: 2435-1), GSK-3β (Santa Cruz Biotechnology, Santa Cruz, CA, USA; Catalog No: sc-9166), RAGE (GeneTex, Irvine, CA, USA; Catalog No: GTX23611), TLR2 (Abcam; Catalog No: T0337), TLR4 (Abcam; Catalog No: T0324), β-Catenin (BD Transduction Lab., San Jose, CA, USA: Catalog No: 610154), β-actin (Sigma Aldrich; Catalog No: A5441), TGFβ1(LAP) (GeneTex , Catalog No: GTX10630), GADPH (Novus, Littleton, CO, USA; Catalog No: NB300-221), Ki-67 (Bioledgend, CA, USA, Catalog No: 652401) and LRP6 phospho (Cell Signaling Technology, Danvers, MA, USA; Catalog No: 2568). After washing with Tris-buffered saline and Tween 20 (TBST), blots were incubated with horseradish peroxidase-conjugated secondary antibodies (Jackson ImmunoResearch) in TBST and developed using an enhanced chemiluminescence-detection system (PerkinElmer, Waltham, MA, USA).

### Statistical analysis

All values were expressed as mean ± SD (n ≥ 3) and analyzed using one-way analysis of variance, followed by Newman-Keuls post-hoc comparison. A *p* < 0.05 was considered statistically significant.

## Additional Information

**How to cite this article**: Chen, Y.-C. *et al*. High mobility group box 1–induced epithelial mesenchymal transition in human airway epithelial cells. *Sci. Rep.* **6**, 18815; doi: 10.1038/srep18815 (2016).

## Supplementary Material

Supplementary Information

## Figures and Tables

**Table 1:** Genes involved in epithelial-mesenchymal transition in HMGB1 treated primary human bronchial epithelial cells.

Function catagory	Gene symbol	Protein	Fold change
Extracellular matrix and cell adhesion	*OCLN*	occludin	0.44
	*TJP1*	tight junction protein ZO-1	0.49
	*CDH1*	E-cadherin	0.56
	*VIM*	vimentin	2.89
	*LAMA5*	Laminin subunit alpha-5	0.67
	*SDC1*	syndecan-1	0.71
	*DCS2*	DeCapping Scavenger2	0.68
	*ILK*	integrin-linked kinase	1.71
	*SPARC*	secreted protein, acidic, cysteine-rich (osteonectin)	2.88
	*KRT19*	keratin 19	1.86
	*KRT14*	keratin 14	1.57
	*FN1*	Fibronectin 1	1.86
Differentiation and development	*FZD7*	frizzled-7	0.51
	*TCF4*	transcription factor 4	1.45
	*FGFBP1*	fibroblast growth factor binding protein 1	2.94
	*BMP2*	bone morphogenetic protein 2	1.71
	*SNAI2*	snail family zinc finger 2	3.03
	*TMRFF1*	transmembrane protein with EGF-like and two follistatin-like domains 1	1.93
	*NOTCH1*	Notch homolog 1, translocation-associated	1.55
	*TWIST1*	twist family bHLH transcription factor 1	2.23
Migration and motility	*MSN*		2.33
	*SERPINE1*	serpin E1; Plasminogen activator inhibitor-1 (PAI-1)	2.43
	*MMP2*	matrix metallopeptidase 2	2.1
	*MMP9*	matrix metallopeptidase 9	2.0
	*TIMP1*	tissue inhibitor of metalloproteinase 1	1.13
	*PLEK*	pleckstrin	1.82
	*TFPI2*	tissue factor pathway inhibitor 2	2.46
G-protein coupled receptor	*RGS2*	Regulator of G-protein signaling 2	0.76
Pro-inflammatory	*IL1RN*	interleukin-1 receptor antagonist	0.74
	*S100-A4*	S100-A4	1.54
other	*VPS13A*	Vacuolar protein sorting-associated protein 13A	0.69
	*CALD1*	Caldesmon	1.24
