# Supplementary material for: High mobility group box 1-induced epithelial mesenchymal transition in human airway epithelial cells
Source: Sci Rep. 2016 Jan 7;6:18815. doi: 10.1038/srep18815 (PMC4703978; doi:10.1038/srep18815)
Supplement: Supplementary Information [file srep18815-s1.doc]

Supplementary information

**Figure S1. HMGB1 induced EMT in BEAS-2B and HBE cells, as well as increased LRP-6-p but did not increase cell proliferation in BEAS-2B cells.** **(A)** BEAS-2B cells were treated with different doses of HMGB1 for 24 h for protein analysis. Immunofluorescence staining of E-cadherin, ZO-1, and vimentin (green) was detected by fluorescence microscopy. Isotype (negative) control was performed with mouse IgG or rabbit IgG as a primary Ab. Nuclear DNA was stained with DAPI (blue). Images were acquired at ×200 magnification. **(B)** Quantification of immunofluorescence staining using ImageJ software. Data are expressed as mean ± S.D. (n =5) ***p < 0.001 as compared with control group. **(C)** Human bronchial epithelial cell line (HBE cells) treated with HMGB1 for 24h and E-cadherin was detected by immunofluorescence staining and observed by confocal microscopy. **(D)** BEAS-2B cells were treated with different doses of HMGB1 for 24h and LRP-6-p was assessed by western blotting assay. **(E)** BEAS-2B cells were treated with different doses of HMGB1 for 24h and Ki-67 was assessed by western blotting assay. Quantification of protein expression was performed using ImageJ software. Data are expressed as mean ± S.D. (n =3) *p < 0.05 as compared with control group. In immunoblotting assay, gels have been run under the same experimental conditions. Then cropped blots were incubated with different primary antibodies for analysis of signaling pathway.

**Figure S2. Knock-down β-catenin and RAGE impaired HMGB1 activated β-catenin signaling. (A)** Stable β-catenin shRNA BEAS-2B cell clone was treated with HMGB1 (300 ng/mL) for 24 h and protein expression of Snail and Twist was detected by western blotting. **(B)** Stable RAGE shRNA BEAS-2B cell clone was treated with HMGB1 (300 ng/mL) for 24 h and protein expression of β-catenin was detected by western blotting. Quantification of protein expression was performed using ImageJ software. **(C)** The cell morphology of normal, vector (plasmid control), and stable β-catenin shRNA BEAS-2B cell clone were observed by microscopy. **(D)** Stable β-catenin shRNA BEAS-2B cell clone was treated with HMGB1 (300 ng/mL) for 24 h and cell proliferation was detected by BrdU incorporation assay. Data are expressed as mean ± S.D. (n= 3). In immunoblotting assay, gels have been run under the same experimental conditions. Then cropped blots were incubated with different primary antibodies for analysis of signaling pathway.

**Figure S3. Original blots of Fig. 2(b).** Arrows indicated chopping lines.

**
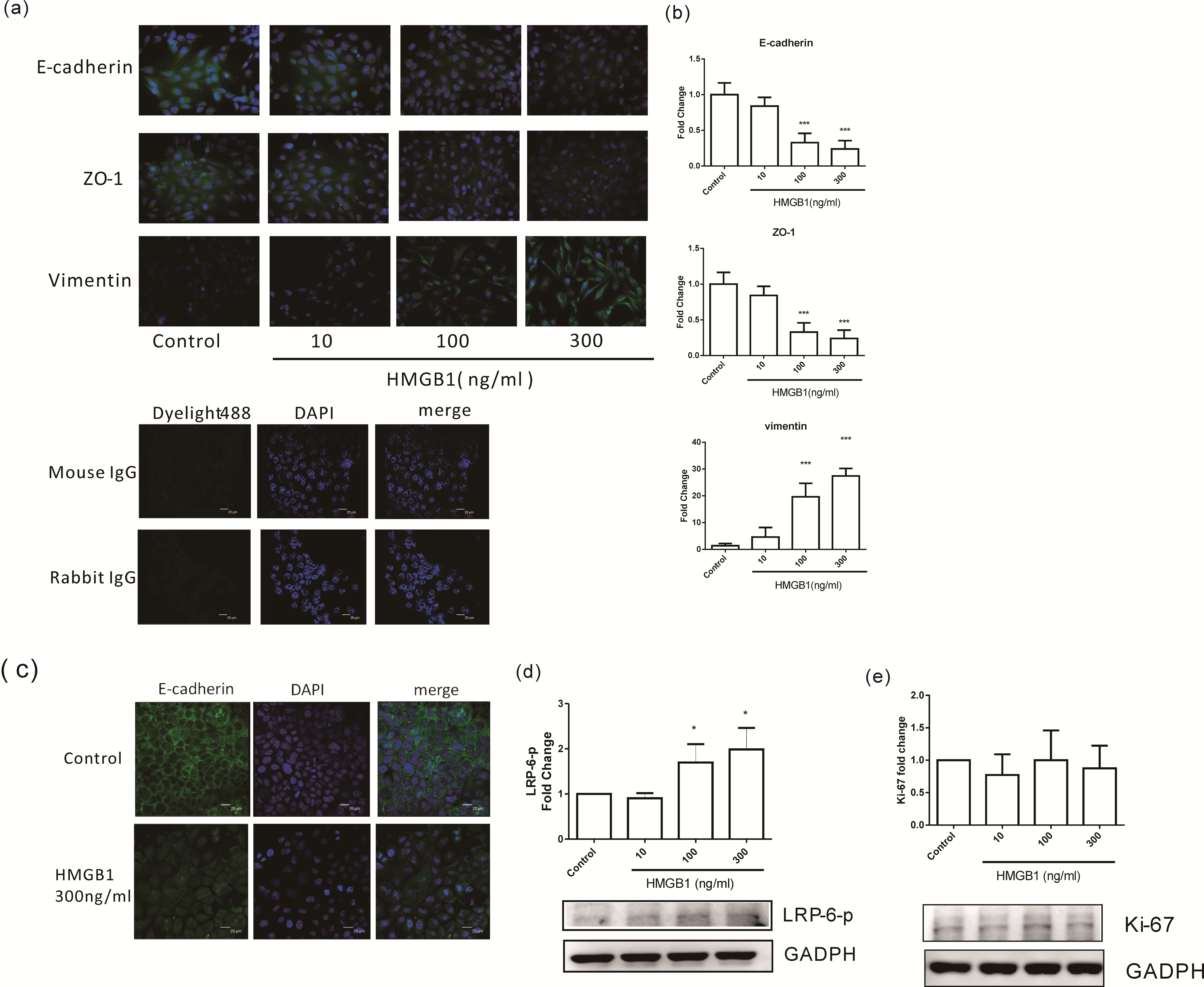
**

**Figure S1**

**
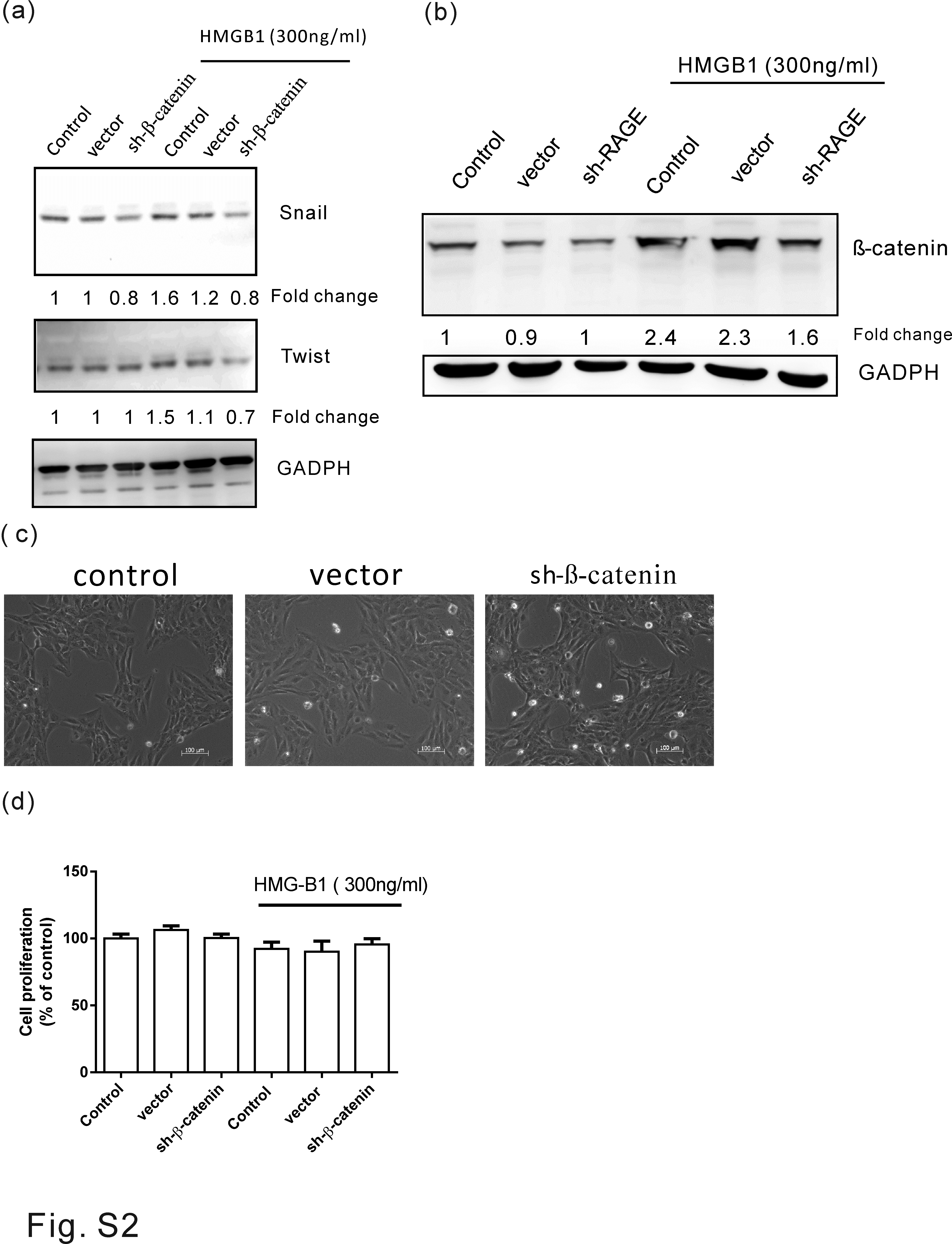
**

**Figure S2.**

**
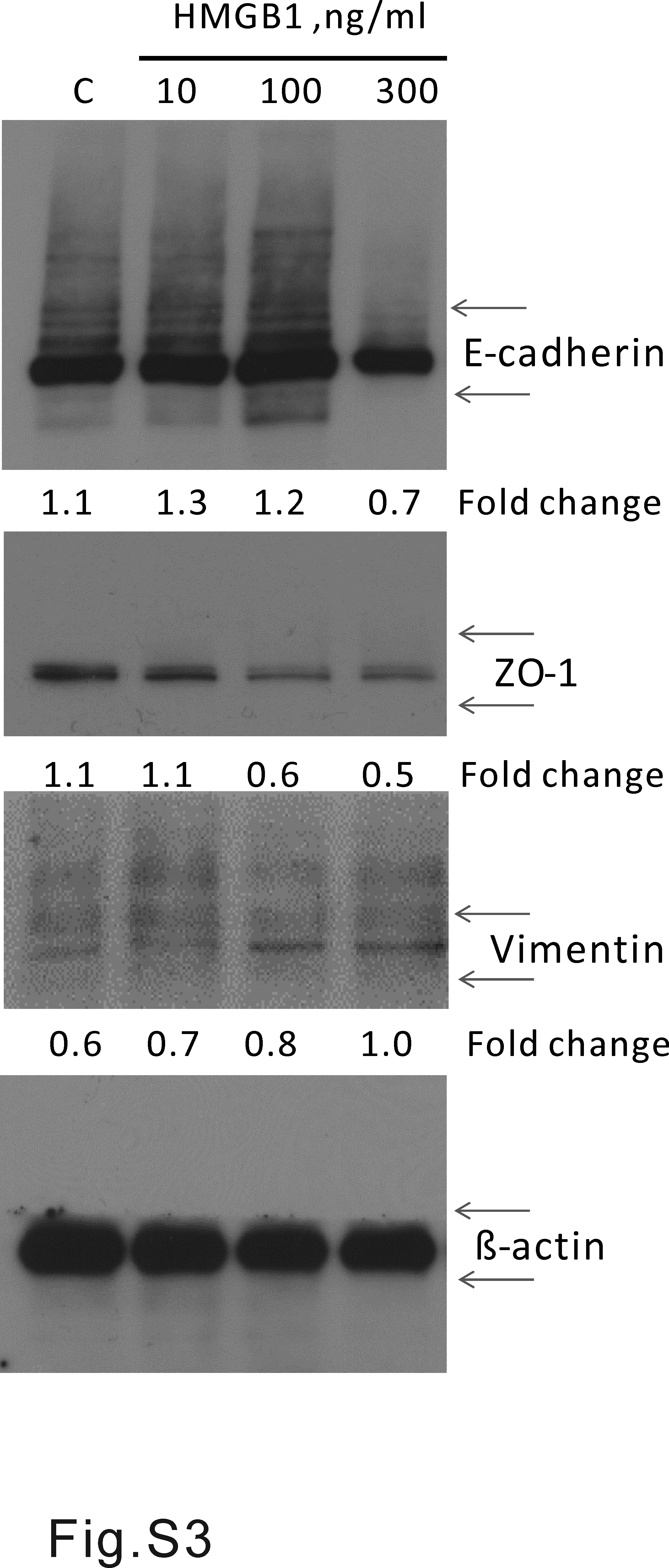
**

**Figure S3.**
